# N-Acetylcysteine Prevents Hypertension via Regulation of the ADMA-DDAH Pathway in Young Spontaneously Hypertensive Rats

**DOI:** 10.1155/2013/696317

**Published:** 2013-12-17

**Authors:** Nai-Chia Fan, Chih-Min Tsai, Chien-Ning Hsu, Li-Tung Huang, You-Lin Tain

**Affiliations:** ^1^Department of Pediatrics, Chang Gung Memorial Hospital at Linkou, Chang Gung University College of Medicine, Taoyuan, Taiwan; ^2^Department of Pediatrics, Kaohsiung Chang Gung Memorial Hospital and Chang Gung University College of Medicine, 123 Dabi Road, Niausung, Kaohsiung 833, Taiwan; ^3^Department of Pharmacy, Kaohsiung Chang Gung Memorial Hospital, Kaohsiung, Taiwan; ^4^Graduate Institute of Clinical Pharmacy, College of Pharmacy, Kaohsiung Medical University, Kaohsiung, Taiwan; ^5^Center for Translational Research in Biomedical Sciences, Kaohsiung Chang Gung Memorial Hospital, Kaohsiung, Taiwan

## Abstract

Asymmetric dimethylarginine (ADMA) reduces nitric oxide (NO), thus causing hypertension. ADMA is metabolized by dimethylarginine dimethylaminohydrolase (DDAH), which can be inhibited by oxidative stress. N-Acetylcysteine (NAC), an antioxidant, can facilitate glutathione (GSH) synthesis. We aimed to determine whether NAC can prevent hypertension by regulating the ADMA-DDAH pathway in spontaneously hypertensive rats (SHR). Rats aged 4 weeks were assigned into 3 groups (*n* = 8/group): control Wistar Kyoto rats (WKY), SHR, and SHR receiving 2% NAC in drinking water. All rats were sacrificed at 12 weeks of age. SHR had higher blood pressure than WKY, whereas NAC-treated animals did not. SHR had elevated plasma ADMA levels, which was prevented by NAC therapy. SHR had lower renal DDAH activity than WKY, whereas NAC-treated animals did not. Renal superoxide production was higher in SHR than in WKY, whereas NAC therapy prevented it. NAC therapy was also associated with higher GSH-to-oxidized GSH ratio in SHR kidneys. Moreover, NAC reduced oxidative stress damage in SHR. The observed antihypertensive effects of NAC in young SHR might be due to restoration of DDAH activity to reduce ADMA, leading to attenuation of oxidative stress. Our findings highlight the impact of NAC on the development of hypertension by regulating ADMA-DDAH pathway.

## 1. Introduction

The imbalance between reactive oxygen species (ROS) and nitric oxide (NO) has been implicated in the pathogenesis of hypertension [1, 2]. Asymmetric dimethylarginine (ADMA), an endogenous inhibitor of nitric oxide synthase (NOS) can reduce NO synthesis while inducing superoxide production, thus playing an important role in the ROS/NO imbalance. ADMA is mainly metabolized by dimethylarginine dimethylaminohydrolase isoforms-1 and -2 (DDAH-1 and -2) in the kidneys and liver [3]. ROS induces ADMA accumulation by inhibiting DDAH, which can be prevented by antioxidants [4, 5].

Glutathione (GSH) is the major intracellular antioxidant [6]. The antioxidant activity of GSH depends mainly on 2 rate-limiting processes: the supply of cysteine and the activity of *γ*-glutamylcysteine synthetase [6]. N-Acetylcysteine (NAC) facilitates intracellular GSH synthesis by increasing the supply of cysteine, the precursor of GSH. Several studies have provided evidence of an impaired GSH system playing a role in hypertension [7–11]. Depletion of GSH increases oxidative stress and blood pressure in normotensive rats [7, 8]. Furthermore, NAC improves NO bioavailability to reduce blood pressure in adult spontaneously hypertensive rats (SHR) [9, 10]. It has also been observed that the components of the GSH system are impaired in young SHR kidneys prior to the development of hypertension [11].

We recently observed that melatonin, a hormone produced by the pineal gland, concurrently prevents the increases in ADMA and hypertension in young SHR [12]. In addition, we noted that the protective effect of melatonin after bile-duct ligation- (BDL-) induced kidney injury is associated with increases in both DDAH activity and the GSH to oxidized glutathione (GSSG) ratio [13]. Given that melatonin has an antioxidant capacity [14], our data suggest that melatonin might restore the reduced GSH/GSSG ratio and ROS-inhibited DDAH activity, thus reducing ADMA levels.

NAC has been reported to reduce ADMA levels in patients on hemodialysis [15]; however, the mechanism is unclear. Therefore, it would be of interest to elucidate whether NAC reduces ADMA via upregulation of DDAH. In the current study, we aimed to examine whether NAC prevents the development of hypertension and ADMA accumulation in SHR and whether NAC regulates the GSH/GSSG ratio and DDAH activity to reduce ADMA.

## 2. Materials and Methods

### 2.1. Animals

This study was carried out in strict accordance with the recommendations in the Guide for the Care and Use of Laboratory Animals of the National Institutes of Health. The protocol was approved by the Institutional Animal Care and Use Committee of the Kaohsiung Chang Gung Memorial Hospital (permit number: 2008030504). All efforts were made to minimize suffering.

Three-week-old male SHR and control normotensive male Wistar Kyoto rats (WKY) were obtained (BioLASCO Taiwan Co., Ltd., Taipei, Taiwan). Rats were housed and maintained in an AAALAC-accredited facility, with free access to tap water and standard rat chow. Rats aged 4 weeks were randomly assigned into 3 groups (*n* = 8 for each group): Group 1, WKY without treatment; Group 2, SHR without treatment; and Group 3 (SHR + NAC), which received 2% NAC in drinking water (2000 mg/kg/day). NAC was purchased from Sigma (St Louis, MO, USA). The dose of NAC used here was based on previous studies conducted in rats [7, 10]. Blood pressure was measured in conscious rats by an indirect tail-cuff method (BP-2000, Visitech Systems, Inc., Apex, NC, USA) at 4, 6, 8, 10, and 12 weeks of age [12]. To ensure accuracy and reproducibility, the rats were acclimated to restraint and tail-cuff inflation for 1 week prior to the experiment, and measurements were taken at 13:00–17:00 h each day. Rats were placed on the specimen platform, and their tails were passed through tail cuffs and secured in place with tape. Following a 10 min warm-up period, 10 preliminary cycles were performed to allow the rats to adjust to the inflating cuff. For each rat, 5 cycles were recorded at each time point. Three stable measures were taken and averaged.

All rats were sacrificed at the age of 12 weeks. Twenty-four-hour urine collections were performed once before sacrifice for determination of NO_*x*_ (NO_2_^−^ + NO_3_^−^) levels by the Griess reaction. For the urine collection, rats were housed individually in metabolic cages (Nalgene, Nalge, Rochester, NY). These cages provide separation of urine and feces with the combination of a collecting funnel and a separating cone in the lower chamber. Rats were acclimated for 24 hours in the cages, followed by 24-hour urine collection. Animals were sacrificed by an i.p. overdose of pentobarbital. Heparinized blood samples were collected and the kidneys and heart were harvested. One kidney was removed and snapped frozen; the other kidney was perfusion fixed and removed for immunohistochemistry. The ratio of renal GSH to oxidized GSH (GSH/GSSG) was measured using a commercial kit (Glutathione Assay Kit; Calbiochem, La Jolla, CA, USA) as described previously [13].

### 2.2. Detection of L-Arginine and ADMA by High-Performance Liquid Chromatography

Plasma and tissue L-arginine and ADMA levels were measured by high-performance liquid chromatography (HP series 1100; Agilent Technologies Inc., Santa Clara, CA, USA) with the *o*-phthaldialdehyde/3-mercaptopropionic acid (OPA-3MPA) derivatization reagent, as described previously [12]. The standards contained L-arginine and ADMA at 1–100 *μ*M and 0.5–5 *μ*M, respectively. The recovery rate was 85% to 105%. The tissue concentration was adjusted for protein concentration and expressed as *μ*M/mg protein.

### 2.3. Western Blot

Western blot analysis was performed as described previously [16]. We used the following antibodies: for nNOS-*α*, mouse monoclonal antibody (Santa Cruz); for nNOS-*β*, a rabbit polyclonal antibody (Affinity BioReagents, Golden, CO, USA); for eNOS, a mouse monoclonal antibody (1 : 250, 1-hour incubation; Transduction Laboratories); for DDAH, goat anti-rat DDAH-1 (1 : 500, overnight incubation; Santa Cruz, Santa Cruz, CA, USA) and goat anti-rat DDAH-2 (1 : 100, overnight incubation; Santa Cruz). The bands of interest were visualized using ECL reagents (PerkinElmer, Waltham, MA, USA) and quantified by densitometry (Quantity One Analysis software; Bio-Rad), calculated as the integrated optical density (IOD) minus the background value. The IOD was adjusted for Ponceau red staining (PonS) to correct for variations in total protein loading. Protein abundance was expressed as IOD/PonS.

### 2.4. DDAH Activity

DDAH activity was analyzed by a colorimetric assay measuring the rate of citrulline production, as recently optimized by us [17]. Renal homogenate was incubated with urease for 15 min, and then 100 *μ*L (2 mg) of homogenate was incubated with 1 mM ADMA for 45 min at 37°C. After deproteinization, supernatant was incubated with color mixture at 60°C for 110 min. The absorbance was measured by spectrophotometry at 466 nm. The DDAH activity was represented as *μ*M citrulline/g protein/min at 37°C. To further understand whether GSH and NAC regulate renal DDAH activity, we incubated homogenates of WKY or SHR kidney with NAC (0.1 mM, 10 mM) or GSH (0.25 mM, 2.5 mM) for 30 min and then measured DDAH activity.

### 2.5. Detection of Superoxide by EPR

Superoxide production was measured by electron paramagnetic resonance (EPR) spectroscopy with hydroxylamine spin probe 1-hydroxy-3-carboxypyrrolidine (CPH), as we previously described [16]. Kidney homogenate was prepared, and 10 *μ*g of protein was added to 1 mM CPH and 0.1 mM diethylenetriaminepentaacetic acid in a total volume of 100 *μ*L of Chelex-treated phosphate-buffered saline (PBS). Samples were placed in a 50 *μ*L glass capillary (Wilmad Glass, Buena, NJ, USA). The EPR spectra were recorded using an EMX Plus EPR spectrometer (Bruker Biospin, Rheinstetten, Germany) equipped with an EMX-m40X microwave bridge operating at 9.87 GHz.

### 2.6. Immunohistochemistry Staining for 8-OHdG

Paraffin-embedded tissue sectioned at 4 *μ*m thickness was deparaffinized in xylene and rehydrated in a graded ethanol series to phosphate-buffered saline. Immunohistochemical staining was performed using anti-8-hydroxydeoxyguanosine (8-OHdG) antibody (1 : 2500, Santa Cruz) with a super sensitive polymer-horseradish peroxidase (HRP) IHC detection kit (BioGenex, San Ramon, CA, USA), as we described previously [12]. Identical staining omitting incubation with primary antibody was used as a negative control.

### 2.7. Statistics

First, the Shapiro-Wilk normality test was used to determine which data were normally distributed. Normally distributed data are given as mean ± S.E.M. For most parameters, statistical analysis was done using 1-way ANOVA with Tukey's post hoc test for multiple comparisons. Blood pressure and DDAH activity were analyzed by 2-way repeated-measures ANOVA and Tukey's post hoc test. A *P* value < 0.05 was considered statistically significant. All analyses were performed using the statistical package for the social sciences (SPSS) software.

## 3. Results

**Figure 1:**
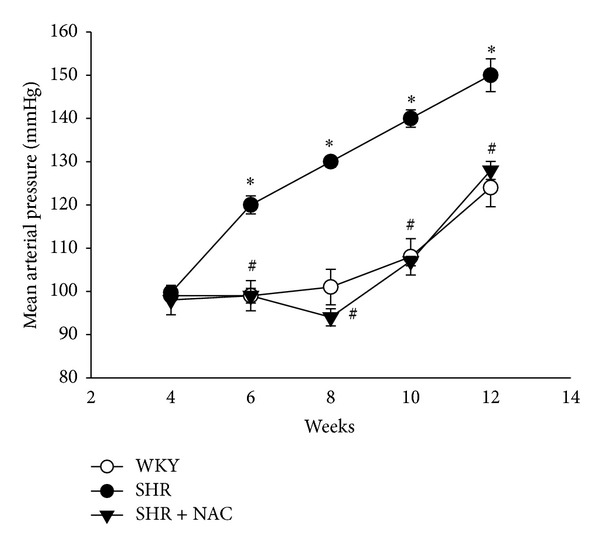
Effects of NAC on mean arterial pressure in SHR and control WKY rats. SHR+NAC, spontaneously hypertensive rat treated with N-acetylcysteine; *n* = 8 per group. *P < 0.05 SHR versus WKY; ^#^ *P* < 0.05  SHR + NAC versus SHR.

After 8 weeks of experiment, the body weight (BW) was lower in the SHR + NAC group compared to the SHR group (Table 1). The heart-weight-to-BW ratio and kidney-weight-to-BW ratio did not differ among the 3 groups. As shown in Figure 1, the systolic blood pressure of SHR was significantly greater than that of age-matched WKY from ages 8 to 12 weeks, during the development of hypertension. This increase in blood pressure was prevented by NAC therapy. In addition, urinary NO_*x*_ (NO_2_^−^ + NO_3_^−^) levels were lower in untreated SHR than in WKY rats, but this difference was not seen in SHR receiving NAC.

As shown in Table 2, ADMA levels in the plasma were higher in SHR than in the WKY group. NAC therapy partially prevented the increase in ADMA levels in SHR. Since ADMA and L-arginine compete for NOS, the arginine-to-ADMA ratio has been used to represent NO bioavailability. Untreated SHR showed a lower arginine-to-ADMA ratio in the plasma than did WKY, but this difference was not found in rats receiving NAC therapy. In the kidneys, SHR receiving NAC had lower renal L-arginine and ADMA levels than untreated SHR. However, the arginine-to-ADMA ratio in the kidneys did not differ among the 3 groups.

**Figure 2:**
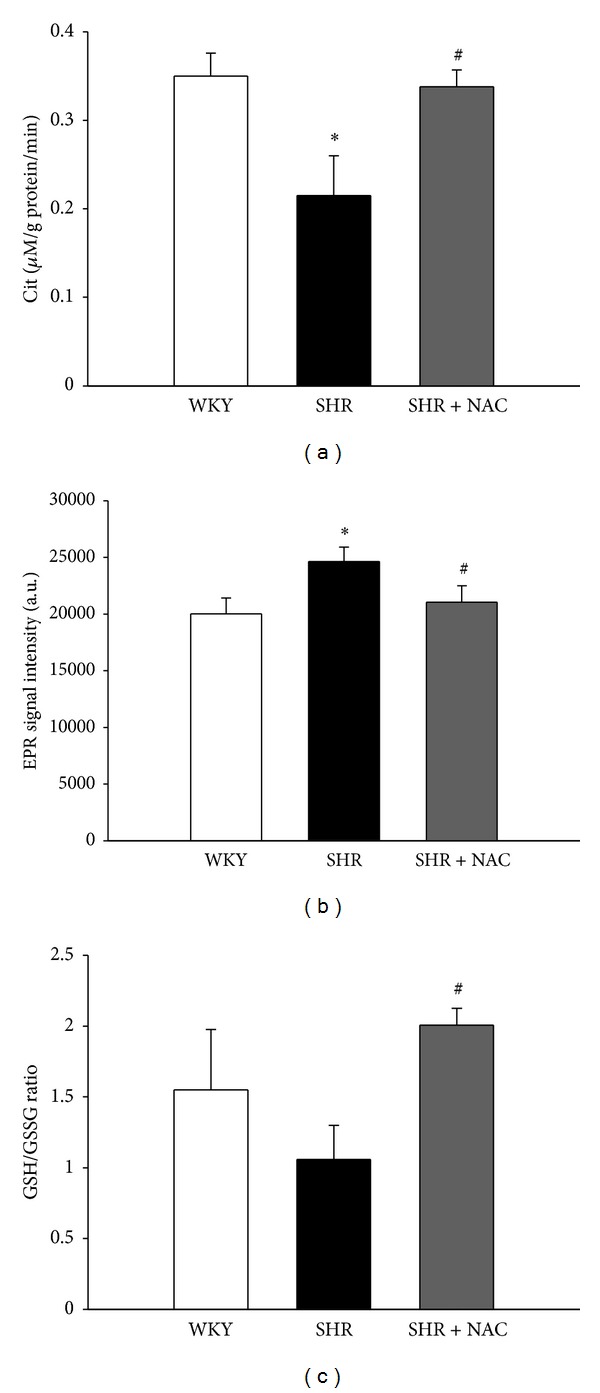
Effects of NAC on renal DDAH activity, superoxide production, and GSH/GSSG ratio. (a) Renal DDAH activity detected by a colorimetric assay; (b) superoxide production in the kidney detected by electron paramagnetic resonance (EPR); (c) renal GSH/GSSG ratio; *N* = 5 per group. *P < 0.05 SHR versus WKY; ^#^ *P* < 0.05 SHR + NAC versus SHR.

**Figure 3:**
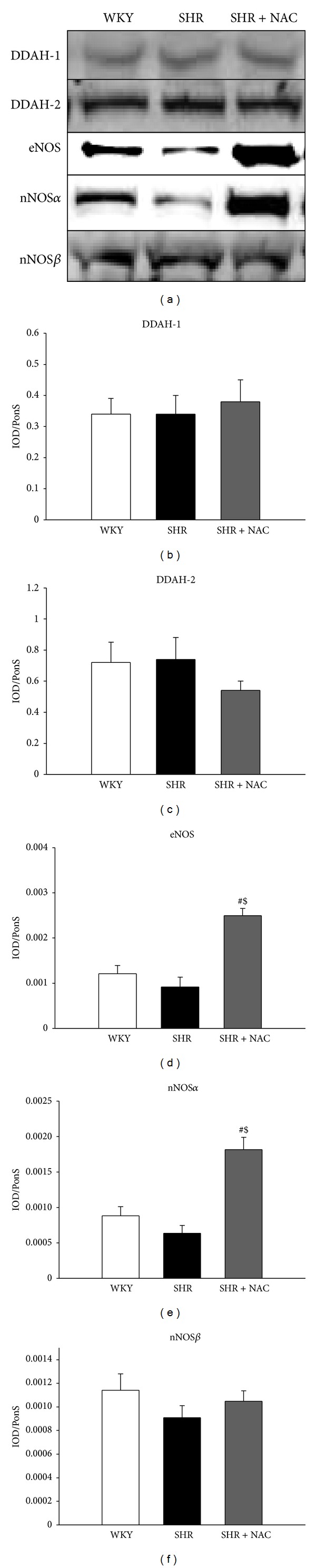
Protein levels of NOS enzymes in SHR and control WKY rats. Representative western blots (a) showed DDAH-1 (34 kDa), DDAH-2 (30 kDa), eNOS (150 kDa), nNOS-*α* (160 kDa), and nNOS-*β* (140 kDa) bands in WKY rats and SHR at 12 weeks of age. Relative abundance of renal cortical (b) DDAH-1, (c) DDAH-2, (d) eNOS, (e) nNOS-*α*, and (f) nNOS-*β* as quantified. SHR + NAC, spontaneously hypertensive rat treated with N-acetylcysteine; *n* = 8/group; *P < 0.05 SHR versus WKY; ^#^ *P* < 0.05 SHR + NAC versus SHR.

**Figure 4:**
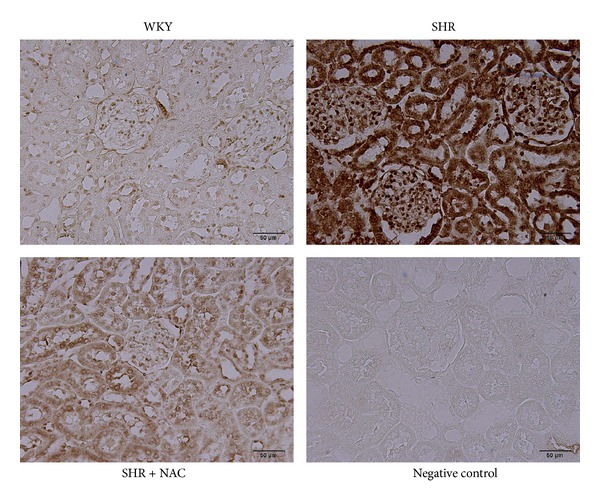
Effects of NAC on 8-OHdG immunostaining in SHR and control WKY rats. Light micrographs illustrating immunostaining for 8-OHdG in the kidney in control WKY (upper left panel), spontaneously hypertensive rats (SHR) (upper right panel), N-acetylcysteine-treated SHR (SHR + NAC) (lower left panel), and negative control (lower right panel) at 12 weeks of age. Data are representative of 6 independent rats per group with similar trends. Scale bar: 50 *μ*m.

We next studied the activity and expression of proteins involved in the ADMA pathway. As in our previous study [12], we found that renal DDAH activity was lower in SHR than in WKY rats. As shown in Figure 2(a), decreased renal DDAH activity in SHR was prevented by NAC therapy. Next, we determined superoxide production in the kidneys using EPR spin trapping. We found that the changes in superoxide production (Figure 2(b)) in the 3 groups were quite similar to the changes in plasma ADMA levels (Table 2). Renal superoxide production was higher in SHR than in the WKY group, whereas NAC therapy prevented the increase in superoxide in SHR kidneys. In contrast, NAC significantly increased the renal GSH/GSSG ratio in SHR kidneys (Figure 2(c)). We found no differences in ADMA-metabolizing enzyme, DDAH-1, and DDAH-2 abundances (Figures 3(a)–3(c)) among the 3 groups. Renal cortical eNOS protein expression (Figure 3(d)) was higher in the SHR + NAC group than that in WKY, as was renal nNOS-*α* protein abundance (Figure 3(e)). There was no significant difference in nNOS-*β* protein abundance among the 3 groups (Figure 3(f)). As shown in Figure 4, the staining intensities of 8-OHdG in SHR were apparently stronger in both glomeruli and renal tubules than those in WKY. The SHR + NAC group revealed intermediate staining of 8-OHdG.

**Figure 5:**
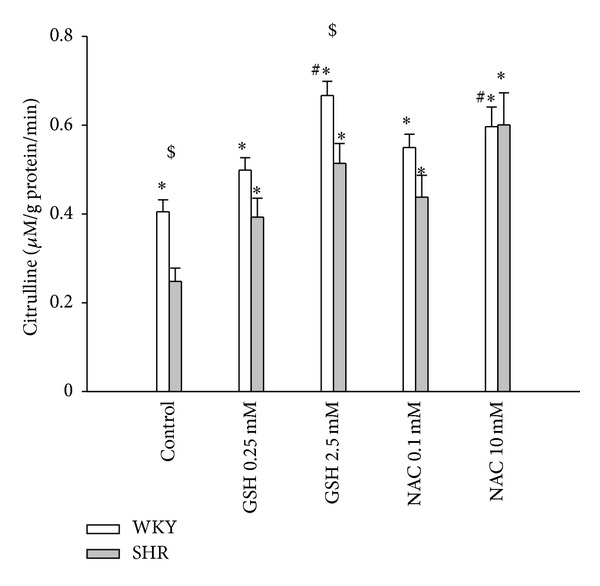
Effects of NAC and GSH on renal DDAH activity. Posttranslational effect of N-acetylcysteine (NAC) and glutathione (GSH) on renal DDAH activity in WKY rats and SHR. WKY and SHR kidney homogenates were treated with different concentrations of GSH (0.25 mM, 2.5 mM) or NAC (0.1 mM, 10 mM). All measurements were made in triplicate. *P < 0.05 versus untreated SHR; ^#^ *P* < 0.05 versus untreated WKY; ^$^ *P* < 0.05 SHR versus WKY with the same treatment.

To further understand whether the means by which NAC regulates renal DDAH activity is related to GSH, we incubated homogenates of WKY or SHR kidney with different concentrations of NAC or GSH in vitro. Our results demonstrated that both NAC and GSH similarly restore decreased renal DDAH activity in SHR, reaching the same level as in WKY. With high concentrations, NAC and GSH both increased renal DDAH activity in SHR as well as in WKY (Figure 5).

## 4. Discussion

The major findings of this study are as follows: (1) NAC treatment attenuates hypertension development in young SHR; (2) NAC administration results in a reduction in plasma ADMA levels, a decrease in superoxide production, and an increase in DDAH activity and the GSH/GSSG ratio in the kidneys of SHR; and (3) NAC and GSH both increase renal DDAH activity in vitro.

SHR develop hypertension at 12 weeks of age. This is associated with an elevated ADMA level and a decreased arginine-to-ADMA ratio in the plasma, a decreased urinary NO_*x*_ level, and increased superoxide production in the kidneys. These findings support a link between reduced NO bioavailability, increased oxidative stress, and the development of hypertension, which is in agreement with other experimental and clinical studies [1, 9–11]. In line with an earlier study [10], we found that administration of NAC attenuated the increase in blood pressure occurring in young SHR. Our data demonstrated that several mechanisms may be involved in the process by which NAC restores NO bioavailability to prevent hypertension in SHR: a reduction in plasma ADMA level, restoration of the arginine-to-ADMA ratio in the plasma, increased renal protein levels of eNOS and nNOS-*α*, an increased renal GSH/GSSG ratio, and attenuated oxidative stress.

ADMA plays an important role in the NO/ROS imbalance, contributing to hypertension by stimulating ROS and reducing NO [1]. Our previous report demonstrated that an increase in ADMA level in the plasma and kidneys develops early on, even before the onset of hypertension in SHR [18]. In this study, we first reported that the antihypertensive effect of NAC is associated with its ADMA-lowering effect. NAC has been reported to increase tissue GSH concentrations in SHR [10, 19]. Our previous study suggested that the GSH/GSSG ratio is more representative of the cellular oxidative stress status than are total GSH levels [20]. In this study, therefore, we determined the GSH/GSSG ratio to represent the status of oxidative stress. We found that this ratio was increased in the NAC-treated SHR kidney, suggesting that NAC might increase intracellular GSH synthesis to reduce oxidative stress and consequently attenuate hypertension. A recent study showed that glutamylcysteine, a precursor of GSH, has a strong association with ADMA [21]. Whether this reflects a direct effect of GSH on DDAH activity to reduce ADMA remains unclear. Our study showed for the first time that NAC increased DDAH activity to reduce the ADMA level.

We found no difference in DDAH-1 and -2 protein abundance in the kidneys among the 3 groups. Nevertheless, both NAC and GSH in vitro increased renal DDAH activity at the posttranslational level. In addition to being a substrate for GSH synthesis, other mechanisms of NAC action have been recognized as reducing oxidative stress, including scavenging of radicals and reducing protein disulfides [22]. We recently found that ROS induces ADMA accumulation by inhibiting DDAH activity [4], which can be prevented by antioxidant therapy [12]. Redox modification of a sulfhydryl group in the catalytic region of DDAH could confer reversible sensitivity of the enzyme to oxidative stress [5]. Thus, it is possible that NAC prevented decreased DDAH activity by reducing a sulfhydryl group in DDAH to form a mixed disulfide. In addition, a previous study showed that the increased ROS production induced by ADMA was inhibited by NAC in vitro [23]. Our data are consistent with a previous report [24] in demonstrating that NAC therapy reduced superoxide production; we also found that it attenuated oxidative stress damage (represented by 8-OHdG) in the SHR kidney. Therefore, more than a precursor of GSH, NAC, can restore DDAH activity and reduce ADMA in SHR by a complex redox regulation.

Next, we found that NAC significantly increased protein levels of eNOS and nNOS-*α* in the SHR kidney. Our data support previous studies, showing that NAC not only increased the bioactivity of NO, but also enhanced expression of the eNOS enzyme [10, 25]. A previous study found that NAC induced an increase in nNOS activity in the SHR brain [26]. Importantly, we also found that the NAC-enhanced renal nNOS-*α* protein level increased NO levels (represented by urinary NO_*x*_ level) in the SHR kidney. To our knowledge, this novel finding has never been reported before. Given that NAC is involved in redox homeostasis and that NOS isoform expression can be regulated by redox signaling, it is possible that NAC reduces ADMA and oxidative stress to upregulate eNOS and nNOS-*α* expression. Further studies are warranted to elucidate the underlying mechanisms via which NAC upregulates the transcription/translation of eNOS and nNOS-*α*. Our results show that NAC therapy could shift the ROS-NO balance towards increased NO bioavailability to prevent hypertension in young SHR. GSH and NAC are both thiol antioxidants. Interestingly, captopril, a thiol containing angiotensin-converting enzyme inhibitor, has been reported to inhibit ADMA accumulation [27]. Given that enhancing the activity of DDAH could be the basis for novel therapeutic strategies for preventing many cardiovascular and kidney diseases with raised levels of ADMA [5], the detailed molecular mechanisms whereby thiol modulates the ADMA-DDAH pathway need to be fully elucidated.

Why does NAC reduce renal L-arginine levels in SHR? The decrease in L-arginine levels in the kidney might be due to increased consumption via other metabolic pathways (e.g., arginase) and increased transport from the kidney into the circulation. A previous report showed that NAC can increase renal arginase activity so as to increase ornithine levels [28]. Thus, it is possible that NAC treatment decreased L-arginine levels by increasing arginase activity in the kidney. Next, NAC has been reported to prevent the sulfhydryl modulation-induced reductions in L-arginine transport in endothelial cells [29]. Another possibility is that NAC increases L-arginine transport out of the kidney, consequently reducing renal concentrations of L-arginine.

In conclusion, NAC treatment attenuates the development of hypertension in young SHR, which is correlated with a reduction in plasma ADMA levels, a decrease in superoxide production, and an increase in DDAH activity and GSH/GSSG ratio in the SHR kidney. NAC and GSH both increase renal DDAH activity in vitro. These observations strongly suggest that an increase in renal DDAH activity and a decrease in ADMA might be responsible for the blood pressure-lowing effect of NAC in young SHR. NAC therapy might restore the ROS-NO balance, thus preventing the development of hypertension. Our findings highlight the therapeutic potential of NAC with respect to the ADMA-DDAH pathway.

## Figures and Tables

**Table 1:** Weights and functional parameters.

	WKY	SHR	SHR + NAC
Body weight (g)	294.6 ± 4.0	308.1 ± 4.5	286.9 ± 3.4^#^
Heart weight (g)	1.16 ± 0.06	1.38 ± 0.03*	1.20 ± 0.03^#^
Heart weight per 100 g body weight	0.39 ± 0.02	0.45 ± 0.01*	0.42 ± 0.01
Left kidney weight (g)	1.19 ± 0.05	1.36 ± 0.07	1.25 ± 0.04
Left kidney weight per 100 g body weight	0.40 ± 0.02	0.44 ± 0.02	0.43 ± 0.01
UNO_*x*_V (*μ*mole·24 hr^−1^·100 g BW^−1^)	1.16 ± 0.21	0.34 ± 0.15*	0.69 ± 0.11^#^

SHR + NAC, spontaneously hypertensive rat treated with N-acetylcysteine; *n* = 8 per group; UNO_*x*_V, total urinary NO_*x*_ (NO_3_^−^ + NO_2_^−^) excretion. *P < 0.05 SHR versus WKY; ^#^ *P* < 0.05 SHR + NAC versus SHR.

**Table 2:** Plasma and tissue levels of L-arginine, ADMA, and arginine-to-ADMA ratio.

	WKY	SHR	SHR + NAC
Plasma (*μ*mole/L)			
L-arginine	137.1 ± 7.3	140.1 ± 3.3	134.4 ± 4.5
ADMA	1.14 ± 0.1	1.68 ± 0.11*	1.37 ± 0.16
Arginine-to-ADMA ratio	125 ± 14	85 ± 6*	104 ± 11
Kidney (*μ*mole/L/mg protein)			
L-arginine	88.1 ± 15.1	87.1 ± 12.3	43.3 ± 6.0^#$^
ADMA	2.53 ± 0.38	2.15 ± 0.34	1.11 ± 0.17^#$^
Arginine-to-ADMA ratio	36 ± 6	45 ± 9	40 ± 3

SHR + NAC, spontaneously hypertensive rat treated with N-acetylcysteine; *n* = 6 per group; *P < 0.05 SHR versus WKY; ^#^ *P* < 0.05 SHR + NAC versus SHR; ^$^ *P* < 0.05 SHR + NAC versus WKY.
